# Digital Approaches for Managing Brain Fog in Myalgic Encephalomyelitis/Chronic Fatigue Syndrome (ME/CFS): Interventions, Monitoring, and Future Directions

**DOI:** 10.3390/life16040571

**Published:** 2026-04-01

**Authors:** Diana Araja, Modra Murovska, Angelika Krumina, Ajandek Eory, Uldis Berkis

**Affiliations:** 1 Institute of Microbiology and Virology, Riga Stradins University Research Center, Riga Stradins University, 5 Ratsupites Str., LV-1067 Riga, Latvia; modra.murovska@rsu.lv; 2 Department of Infectology, Riga Stradins University, 16 Dzirciema Str., LV-1007 Riga, Latvia; angelika.krumina@rsu.lv; 3 Division of Integrative Medicine, Department of Family Medicine, Semmelweis University, 7-9 Stahly Str., H-1085 Budapest, Hungary; eory.ajandek@semmelweis.hu; 4 Department of Development and Projects, Riga Stradins University, 16 Dzirciema Str., LV-1007 Riga, Latvia; uldis.berkis@rsu.lv

**Keywords:** myalgic encephalomyelitis/chronic fatigue syndrome (ME/CFS), cognitive impairment, brain fog, digital health, digital biomarkers, cognitive assessment, remote monitoring, biopsychosocial framework, patient-centered care

## Abstract

Myalgic encephalomyelitis/chronic fatigue syndrome (ME/CFS) is a high-burden, under-researched condition characterized by heterogeneous and fluctuating symptoms, including cognitive dysfunction commonly described by patients as “brain fog”. Despite growing interest in digital health technologies for symptom monitoring and personalized care, their application to the assessment and management of cognitive dysfunction in ME/CFS remains unclear. This descriptive review aimed to examine the current scientific evidence on digital approaches related to brain fog in ME/CFS. A structured literature search following PRISMA guidance was conducted to identify relevant studies. The available literature remains limited in scope and methodological maturity. During synthesizing across studies, three main functional domains of digital application become apparent: (1) digital tools for cognitive assessment, which have the strongest evidence base; (2) digital platforms for longitudinal monitoring; and (3) digitally mediated interventions or rehabilitation approaches, both of which are less well studied. Simultaneously, the findings suggest that patient-reported brain fog may represent a visible component of the broader ME/CFS disease spectrum and could serve as an early clinical indicator guiding diagnosis and management. Interpreting these symptoms within a biopsychosocial framework may facilitate understanding of the complex nature of the disease and optimize the use of digital technologies for monitoring cognitive dysfunction and supporting patient-centered care in ME/CFS.

## 1. Introduction

**Myalgic encephalomyelitis/chronic fatigue syndrome (ME/CFS)** is recognized as a high-burden yet under-researched medical condition, as highlighted by the European Commission in a dedicated discussion paper entitled “Scoping study on evidence to tackle high-burden under-researched medical conditions” [1]. ME/CFS is a complex, debilitating disorder characterized by profound fatigue, post-exertional malaise (PEM), unrefreshing sleep, and a range of cognitive symptoms collectively referred to as “brain fog”. Brain fog encompasses difficulties with attention, memory, processing speed, and executive function, significantly impairing daily functioning and quality of life for patients. Prevalence estimates vary, but ME/CFS affects millions worldwide, with a substantial proportion experiencing persistent cognitive dysfunction as a core symptom [2].

Despite decades of investigation, ME/CFS still lacks universally accepted diagnostic **biomarkers**. Numerous potential biomarkers have been proposed; however, their diagnostic performance, methodological quality, and clinical translatability remain highly variable and largely unvalidated [3]. Simultaneously, a recent systematic review of digital biomarkers of fatigue across chronic diseases reported that, among the digital measures assessed, only *activity counts* have been consistently investigated, underscoring the limited development and validation of digital biomarkers relevant to fatigue and ME/CFS [4].

**Diagnostics** are mostly clinical and often by means of exclusion, based on consensus criteria rather than objective tests. The Canadian Consensus Criteria, which are widely used to diagnose ME/CFS, define Neurological/Cognitive Manifestations as one of the criterion categories for this diagnosis [5]. Two or more of the following difficulties should be present: confusion; impairment of concentration and short-term memory consolidation; disorientation; difficulty with information processing, categorizing, and word retrieval; and perceptual and sensory disturbances—e.g., spatial instability and disorientation and inability to focus vision. Ataxia, muscle weakness, and fasciculations are common. There may be overload phenomena—cognitive, sensory (e.g., photophobia and hypersensitivity to noise), and/or emotional overload—which may lead to “crash” periods and/or anxiety [5].

These cognitive deficits form one of the central components of the clinical syndrome and correlate with functional impairment and reduced quality of life in ME/CFS cohorts. A systematic review and meta-analysis of neuropsychological performance consistently demonstrated deficits in immediate memory, information processing speed, and attentional capacity in ME/CFS patients compared to healthy controls, supporting the notion that central nervous system dysfunction contributes to symptomatology beyond subjective reporting alone [6]. Meanwhile, a systematic review of neurological impairments in ME/CFS using neuroimaging techniques revealed that the outcomes from the examined articles included changes in gray and white matter volumes, cerebral blood flow, brain structure, sleep, electroencephalography activity, functional connectivity, and cognitive function [7].

**Neuroinflammatory processes** have emerged as a prominent potential mechanism mediating cognitive symptoms and brain fog in ME/CFS. Positron emission tomography studies using markers of activated microglia (for example, translocator protein binding) have shown elevated neuroinflammation in widespread brain regions in ME/CFS patients compared to healthy controls, with the degree of signal correlating with the severity of cognitive dysfunction and fatigue sensation [8]. These findings align with those of broader neuropathological studies indicating **blood–brain barrier compromise and glial activation**, whereby infiltrating peripheral inflammatory mediators and chronic immune activation may sustain central inflammatory states that impair neuronal function and contribute to cognitive deficits [9]. Elevated levels of proinflammatory cytokines such as interleukin-1β, tumor necrosis factor-α, and interleukin-6 have been implicated in glial activation and neuroimmune crosstalk, capable of disrupting neurotransmitter systems, altering synaptic signaling, and contributing to the subjective experience of mental fatigue [10].

Compounding this neuroinflammatory milieu is **mitochondrial dysfunction**, which directly undermines the energy supply required for sustained cognitive activity. Mitochondria serve as the primary cellular adenosine triphosphate (ATP) generators, and perturbations in mitochondrial metabolism have been reported in ME/CFS, including impaired oxidative phosphorylation and altered energy metabolite profiles [11]. These metabolic inefficiencies likely affect **high-energy-demand regions** of the brain, where insufficient ATP availability can compromise neuronal signaling, synaptic plasticity, and the capacity to sustain cognitive operations under stress or post-exertion. Consistent with this concept, magnetic resonance spectroscopy data have demonstrated **elevated lactate** and other markers indicative of energetic stress in brain regions of ME/CFS patients, a pattern reflecting disrupted energy metabolism that may underpin both physical and cognitive fatigue [12].

Research on the neural architecture underlying cognitive processing further reveals **altered functional connectivity** in ME/CFS, particularly within intrinsic brain networks subserving attention, memory, and executive function. Functional magnetic resonance imaging (fMRI) studies have documented aberrant connectivity between key networks such as the **default mode network** and **salience network**, which are implicated in internally directed cognition, attentional switching, and cognitive control [13]. Furthermore, ultra-high-field MRI investigations have reported **reduced connectivity within ponto-cerebellar regions** and between the cerebellum and cerebral cortex, areas integral to cognitive coordination, motor control, and integration of cognitive and physiological signals [14]. These connectivity alterations may reflect inefficient neural signaling and network integration, consistent with reports of slowed cognitive processing and difficulties sustaining attention in ME/CFS.

Interplay among these mechanisms suggests that **neuroinflammation, metabolic insufficiency, and network dysfunction collectively drive brain fatigue** in ME/CFS. Chronic inflammation may perpetuate glial activation and cytokine release, which, in turn, can interfere with mitochondrial function and disrupt neural circuits essential for cognitive performance. Neuroinflammation can also alter regional cerebral blood flow and neurotransmitter dynamics, exacerbating metabolic stress and further impairing functional connectivity among neural networks [15]. The interaction of these processes may also contribute to the heterogeneity in clinical presentation and the variability in objective findings across studies, complicating efforts to establish definitive biomarkers.

Simultaneously, **interoception** plays an additional role in linking these biological alterations to the subjective experience of brain fatigue in ME/CFS. Interoceptive signaling arises from molecular processes involving cytokine activity, metabolic intermediates, autonomic neurotransmission, and neuroendocrine mediators, which collectively inform predictive brain models responsible for regulating physiological set points and allostatic balance [16]. In ME/CFS, chronic immune activation, mitochondrial and metabolic insufficiency, and autonomic dysregulation may distort these afferent signals, leading to impaired central integration and inaccurate predictions of bodily capacity. Persistent inflammatory and stress-related signaling—such as altered cytokine profiles or hypothalamic–pituitary–adrenal axis dysfunction—may promote maladaptive metacognitive beliefs and reduced regulatory flexibility [17]. Within this framework, fatigue and cognitive dysfunction can be understood as protective, adaptive responses to a perceived energetic threat, providing a mechanistic bridge connecting molecular dysregulation, altered brain network dynamics, and the lived experience of brain fog in ME/CFS.

Moreover, these biological alterations interface with subjective symptom reports and diagnostic challenges. Cognitive fatigue in ME/CFS is inherently subjective and prone to variability across contexts and individuals, making standardized assessment difficult. The absence of validated biomarkers amplifies diagnostic heterogeneity, with patients often fitting diverse criteria sets that may reflect underlying biological subtypes rather than a single pathological process. This complexity underscores why objective assessment and targeted treatment strategies for brain fog have remained elusive, despite accumulating evidence of central nervous system involvement.

Despite its significant impact on daily functioning, brain fog in ME/CFS **lacks robust direct pharmacological therapies** supported by high-level evidence; thus, **disease management strategies** are primarily supportive and symptom-oriented, guided by clinical experience and extrapolation from related conditions. Present ME/CFS pharmacological management includes immunomodulatory treatments, antioxidant therapies, mitochondrial support, and neuroinflammation intervention [9]. No pharmacological agent is currently approved specifically for brain fog in ME/CFS. Some clinicians trial off-label medications aimed at modulating neurotransmitter systems (e.g., low-dose stimulants, modafinil) or addressing sleep–wake regulation, with variable success and frequent tolerability issues. The issue of cognitive impairment as a symptom accompanying chronic fatigue also remains insufficiently investigated within pharmacovigilance systems [18].

**Non-pharmacological strategies** such as *Cognitive Behavioral Therapy*, *activity pacing*, and *Traditional Chinese Medicine* complement biomedical approaches by alleviating symptom severity and promoting energy conservation [9]. Given the absence of disease-modifying treatments for ME/CFS and the multifactorial nature of brain fog, many patients and clinicians turn to **mind–body and lifestyle-oriented interventions** as adjunctive strategies. These approaches aim to modulate stress responses, autonomic balance, immune activation, and symptom perception, rather than directly targeting a single biological pathway. While the evidence quality varies and robust randomized controlled trials are limited, several interventions have demonstrated potential benefits for cognitive symptoms and overall well-being when carefully adapted to the constraints of ME/CFS [19].

Low-intensity, adaptive movement practices such as *gentle yoga, Tai Chi,* and *Qigong* are sometimes employed to support autonomic regulation, body awareness, and cognitive calm. Unlike graded exercise therapy, these practices emphasize slow, controlled movements, breathing, and relaxation, making them more compatible with ME/CFS when appropriately modified. Preliminary studies suggest potential benefits for fatigue severity, mood, and perceived cognitive function [20], possibly mediated through parasympathetic activation and reduced sympathetic overdrive. However, the evidence remains limited, and these practices should be considered *optional adjuncts* rather than core treatments, requiring individualized adaptation and close symptom monitoring.

These **long-standing diagnostic and therapeutic uncertainties** underscore an urgent need for innovative approaches that can support both clinical care and research in ME/CFS. In parallel with these unmet medical needs, rapid advances in **digital health technologies** have transformed the landscape of chronic disease management. Mobile health applications, wearable sensors, remote monitoring platforms, and digital cognitive tools are increasingly used to capture real-world health data, support self-management, and enable decentralized research. Notably, individuals with ME/CFS are among the early and active adopters of such technologies, frequently using symptom-tracking applications, activity monitors, and online platforms to monitor fatigue, cognitive fluctuations, and post-exertional responses in daily life. These patient-driven practices reflect both the absence of effective clinical tools and the potential value of digital solutions for capturing the dynamic, fluctuating nature of ME/CFS symptoms—particularly cognitive impairment and brain fog, which are difficult to assess using conventional clinical instruments.

However, despite the widespread patient use of digital tools, **it remains unclear to what extent these technologies have been systematically studied, validated, or integrated into scientific research and clinical practice for ME/CFS**. Existing evidence suggests that digital biomarkers of fatigue and cognition are underdeveloped, with most studies focusing narrowly on activity counts and lacking disease-specific validation. This gap between real-world use and scientific evidence highlights a critical need to synthesize existing research, assess methodological quality, and identify areas where digital approaches may meaningfully advance the understanding and management of ME/CFS.

The **aim of this descriptive review** is therefore to provide a structured overview of the existing scientific literature on digital approaches applied to ME/CFS, with a particular focus on tools relevant to the assessment and management of brain fog, and to identify key trends and gaps in this emerging research area. By systematically analyzing the available studies, this work seeks to contextualize the current evidence, identify emerging trends and methodological limitations, and highlight gaps that warrant further investigation. Importantly, this review aims not to evaluate the clinical efficacy of specific interventions but rather to map the landscape of digital solutions and their potential roles in research, symptom monitoring, and supportive care.

The **principal conclusions** of this review indicate that, while digital technologies hold considerable promise for addressing key challenges in ME/CFS—such as symptom heterogeneity, symptom fluctuation, and the lack of objective markers—the current evidence base remains fragmented and limited in scope. Most digital approaches have not been rigorously validated in ME/CFS populations, and cognitive symptoms such as brain fog are particularly underrepresented. Nonetheless, the findings underscore the growing relevance of digital health as a complementary avenue for advancing ME/CFS research and care, and they support the need for more targeted, methodologically robust studies integrating digital tools with clinical and biological frameworks.

## 2. Materials and Methods

Although the term “brain fog” is widely used by patients to describe cognitive dysfunction in ME/CFS, it does not correspond to a single, well-defined neuropsychological construct. A phenomenological framework proposed in the literature characterizes brain fog as involving “subjective cognitive symptoms and objective cognitive impairments relating to mental fatigue; brain fog is considered to be a cognitive subtype of fatigue” [21]. Within this framework, several features relevant to cognitive dysfunction in ME/CFS have been described, including:

- Subtle performance deficits in visuospatial short-term memory, processing speed, verbal memory, and visual memory recall;
- Inconsistent findings across studies evaluating cognitive performance;
- Evidence suggesting that observed performance deficits may be mediated by fatigue;
- Associations between subjective cognitive complaints and depressive symptoms, while objective performance measures often show weaker correlations with these factors [21].

Based on these assumptions, in this review, we conceptualize brain fog within a biopsychosocial framework. While biological mechanisms may create vulnerability to cognitive dysfunction, the experience and functional impact of brain fog are shaped by psychological factors (e.g., cognitive fatigue perception, stress reactivity, attentional resource allocation) and contextual influences (e.g., activity demands, environmental load, digital engagement). Digital health approaches frequently target symptom monitoring, behavioral adaptation, cognitive performance assessment, or functional support rather than underlying pathophysiology. Therefore, for analytic clarity, we operationalize brain fog as a **multidimensional construct** encompassing:

1. Subjective cognitive symptoms reported by patients;
2. Objectively measurable cognitive performance deficits;
3. Neurobiological or physiological correlates.

This conceptual framework was used to guide the identification and classification of relevant studies and digital approaches included in the present review.

### 2.1. Study Design

**Figure 1:**
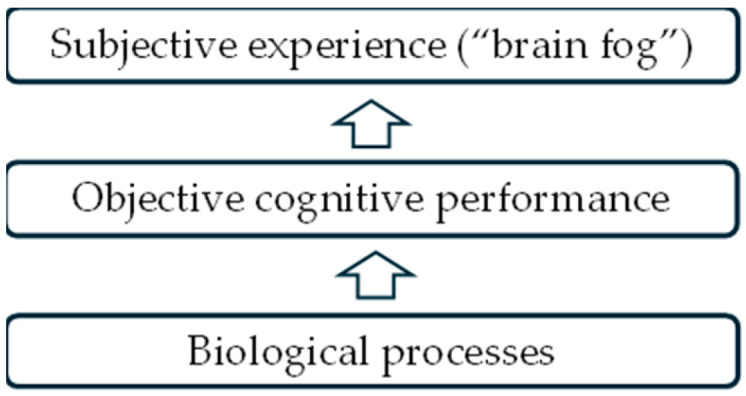
Biopsychosocial conceptualization of brain fog in ME/CFS.

In accordance with this framework (Figure 1), the review examines digital approaches applied to the assessment and management of cognitive dysfunction in ME/CFS across these dimensions.

Brain fog is conceptualized as a multidimensional construct encompassing subjective cognitive symptoms, objectively measurable cognitive performance deficits, and neurobiological or physiological correlates. Digital technologies may contribute to the assessment and monitoring of these dimensions through cognitive testing platforms, neuroimaging analytics, wearable monitoring, and patient-generated data. This framework guided the identification and categorization of digital approaches included in the present review.

### 2.2. Literature Search Strategy

**This descriptive review** was conducted in line with methodological guidance for descriptive reviews [22]. A systematic literature search was carried out across major electronic databases, including Scopus, Web of Science, and PubMed, to identify peer-reviewed publications relevant to the research objective. The search strategy was designed to capture studies addressing digital technologies used for cognitive assessment, monitoring, or intervention in ME/CFS. Search terms were applied to the title, abstract and keywords fields, and were adapted as necessary for each database to account for differences in indexing and search functionality. Search terms included combinations of: (1) condition-related terms, (2) cognition impairment-related terms, and (3) digital health-related terms:

1. “myalgic encephalomyelitis/chronic fatigue syndrome” OR “myalgic encephalomyelitis” OR “chronic fatigue syndrome” OR “ME/CFS”

AND

2. “brain fog” OR “cognitive impairment” OR “cognitive disfunction” OR “cognitive difficulties” OR “cognitive complaints” OR “cognitive fatigue” OR “cognitive interference” OR “cognitive manifestation”

AND

3. digital OR “artificial intelligence” OR “machine learning” OR “deep learning” OR “natural language processing” OR algorithmic OR “predictive modelling” OR “predictive analytic” OR tele OR electronic OR mobile OR apps OR App-based OR wearable OR tracking OR remote OR video OR monitoring OR virtual OR augmented OR ePRO OR platform OR diary OR eHealth OR mHealth OR smart OR informatic OR sensor OR portal OR “self-management tool” OR “decision support system”

Manuscripts published up to 31 December 2025, in English, were considered for inclusion, and no start date restriction was applied. References were identified using the search and selection tools available within the respective databases.

### 2.3. Eligibility Criteria

The inclusion criteria were defined a priori. Eligible studies were required to:

1. Report on original research involving human participants diagnosed with ME/CFS;
2. Investigate digital approaches applied to cognitive assessment, monitoring, management, or analysis related to ME/CFS;
3. Include ME/CFS as a primary condition of interest;
4. Present empirical data derived from patient involvement or from patient-related digital data sources (e.g., Electronic Health Record (EHR)).

Studies were excluded if they:

- Represented grey literature, including theses, conference proceedings, policy documents, reports, or unpublished manuscripts;
- Mentioned ME/CFS only as a secondary or incidental diagnosis;
- Did not involve patients directly in the use of digital solutions, with the exception of large-scale EHR-based analyses;
- Mentioned cognitive impairment only as a general symptom of ME/CFS without being a specific focus of investigation, and if digital solutions were used solely for data processing or analysis rather than for direct assessment, monitoring, or intervention involving patients.

### 2.4. Study Selection Process

All records retrieved from the database searches were compiled, and duplicate entries were removed prior to screening. Titles and abstracts were independently screened by three reviewers to assess eligibility based on the predefined inclusion and exclusion criteria. Articles deemed potentially relevant proceeded to full-text review. Discrepancies between reviewers were resolved through discussion and consensus.

**Figure 2:**
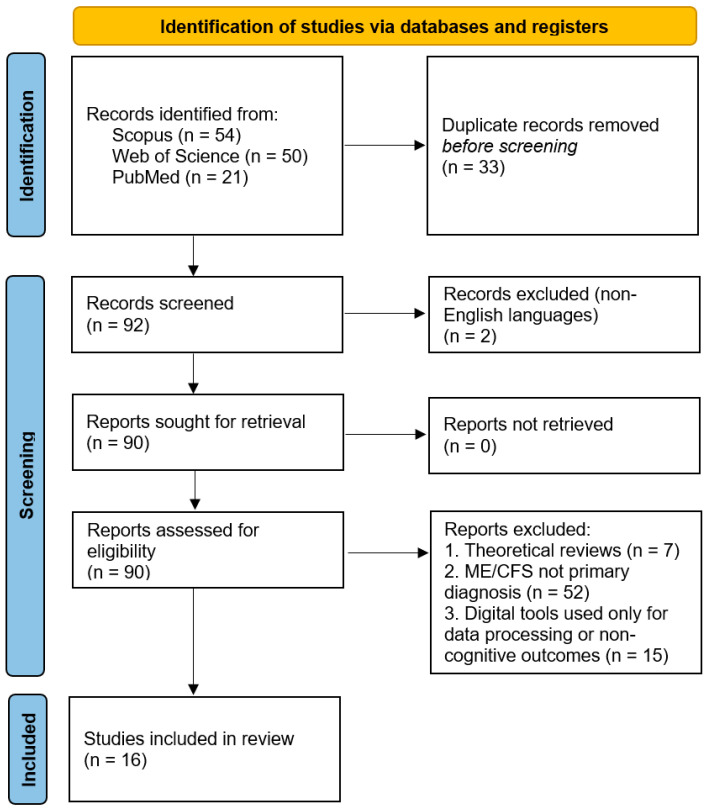
PRISMA flow chart for the identification, screening, and inclusion of records.

The overall study selection process followed the principles of the Preferred Reporting Items for Systematic Reviews and Meta-Analyses (PRISMA) framework [23] and is illustrated in Figure 2.

### 2.5. Data Extraction and Analysis

For each included study, relevant data were systematically extracted and summarized. Particular attention was paid to:

- Research aim;
- Study design;
- Sample size and participant characteristics;
- Types of cognition-related digital solutions employed;
- Key findings, results, and conclusions related to cognitive assessment or management.

A thematic analysis approach was used to synthesize findings across studies. This method enabled the identification of recurring themes, patterns, and gaps in the literature, accommodating the heterogeneity of study designs, digital tools, and outcome measures. The analysis was descriptive in nature, aiming to map the current evidence base rather than to evaluate intervention efficacy quantitatively. A formal assessment of methodological quality or risk of bias was not performed, following the nature of a descriptive review and the ineligibility of grey literature and unpublished manuscripts in the present review. However, risk-of-bias considerations are narratively integrated in Section 4 “Discussion”.

## 3. Results

This review was conducted as a structured descriptive review following PRISMA-informed principles [23] to ensure transparency in search strategy, screening, and study selection. Figure 2 illustrates the article selection process used in this review. The literature search identified a total of 125 records from electronic databases, including Scopus (n = 54), Web of Science (n = 50), and PubMed (n = 21). After the removal of duplicate records (n = 33), 92 unique records were screened based on their titles and abstracts. Two records published in non-English languages were excluded at this stage. The full texts of 90 reports were subsequently assessed for eligibility. Of these, 74 reports were excluded for the following reasons: being theoretical reviews rather than original research (n = 7), not addressing ME/CFS as a primary diagnosis (n = 52), or using digital tools solely for data processing or outcomes unrelated to cognitive impairment (n = 15). Ultimately, 16 studies met the inclusion criteria and were included in the final review.

The results of the evaluation of the selected studies are summarized in Table A1 (Appendix A), including: authors, publication year and reference, research aim, study design, number of patients involved, cognition assessment-related digital solutions, and cognition digital assessment-related results and conclusions of the study. A conceptual classification table (Table 1) provides an overview of digital approaches to cognitive dysfunction in ME/CFS according to cognitive domain, measurement level, and digital function.

Classification of digital approaches to cognitive dysfunction in ME/CFS according to cognitive domain, measurement level, and digital function enables structured analysis.

### 3.1. Overview of Digital Approaches

The 16 included studies demonstrated substantial heterogeneity in both the operationalization of cognitive dysfunction and the function of digital technologies. The digital approaches clustered into five major categories: (1) computerized behavioral cognitive testing, (2) neuroimaging- and biomarker-supported digital analyses, (3) mobile and app-based cognitive assessment, (4) digital interventions targeting cognitive or related symptoms, and (5) data-driven or phenomenological digital approaches (e.g., EHR-based phenotyping and social media analyses).

Across these studies, cognitive outcomes were assessed at three primary measurement levels: subjective self-report, performance-based behavioral testing, and neurobiological correlates. Only a minority of the studies integrated more than one level simultaneously.

### 3.2. Computerized Behavioral Cognitive Testing

The most consistently represented category included computerized neuropsychological testing platforms assessing processing speed, sustained attention, working memory, executive function, and recognition memory. These included early automated telephone adaptations of standardized batteries, web-based testing platforms (e.g., BrainCheck), and smartphone-based reaction time applications.

#### Cross-Study Synthesis

Across these studies, processing speed and sustained attention emerged as the most consistently discriminative domains between ME/CFS patients and controls. Continuous Performance Test paradigms and reaction-time-based assessments demonstrated relatively robust differentiation in cohort studies with adequate sample sizes.

However, several methodological limitations were noted:

- Many of these studies relied on small samples (e.g., pilot cohorts of <50 participants).
- Cross-sectional designs predominated, limiting conclusions about longitudinal fluctuation.
- Few of these studies reported the test–retest reliability in ME/CFS populations.
- Cognitive fatigue during testing was rarely quantified separately from baseline performance.

Despite these limitations, computerized testing appears feasible for remote deployment and scalable assessment. Smartphone-based tools, in particular, show promise for ecological and orthostatic challenge paradigms. However, external validation and standardization across platforms remain limited.

### 3.3. Neurobiological and Machine Learning-Supported Approaches

Several studies used fMRI (BOLD), regional cerebral blood flow measurement (PCASL), or machine learning classification applied to imaging or EHR data. These studies sought to identify objective correlates or computational phenotypes associated with cognitive impairment.

#### Cross-Study Synthesis

The neuroimaging studies provided convergent evidence for altered neural activity or perfusion in regions implicated in attention and limbic regulation. In these studies, machine learning (ML) models demonstrated the ability to differentiate ME/CFS from related conditions (e.g., Gulf War Illness (GWI)) based on multi-regional activation patterns.

However, the following are noted:

- Sample sizes were generally modest.
- Reproducibility across cohorts has not yet been established.
- Clinical applicability remains uncertain.
- Neuroimaging tools are not readily scalable for longitudinal symptom monitoring.

These approaches strengthen biological credibility but are currently better suited for mechanistic research than for routine digital cognitive management.

### 3.4. Mobile and Longitudinal Symptom Monitoring

App-based platforms and multimodal digital trackers enable real-time symptom tracking, sentiment analysis, and integration with multi-omics data. These approaches have primarily assessed subjective cognitive fatigue and global cognitive complaints rather than domain-specific neuropsychological performance.

#### 3.4.1. Strengths

- High ecological validity;
- Longitudinal capability;
- Potential for personalized analytics;
- Feasible integration with wearable data.

#### 3.4.2. Limitations

- Reliance on self-reporting;
- Limited psychometric validation of cognitive-specific measures;
- Often single-case or exploratory designs.

These tools appear particularly suitable for capturing the fluctuating nature of brain fog but require stronger validation frameworks.

### 3.5. Digital Interventions Targeting Cognitive or Related Symptoms

Internet-based Acceptance and Commitment Therapy (ACT), virtual Cognitive Behavioral Stress Management (CBSM), and telerehabilitation programs were used as digital approaches aimed at symptom management.

Although not always directly targeting neurocognitive performance, these interventions addressed psychological stress, somatic distress, and post-exertional symptom exacerbation—factors known to modulate cognitive functioning in ME/CFS.

#### Critical Perspective

- Most of these trials evaluated broader symptom burden rather than specific cognitive domains.
- Cognitive outcomes were often secondary endpoints.
- Evidence for direct improvements in objective neuropsychological performance remains limited.

Nevertheless, these interventions align with a biopsychosocial framework in which cognitive functioning is influenced by stress regulation, activity pacing, and autonomic stability.

### 3.6. Social Media and Virtual Environment Studies

Social media analysis (e.g., of Reddit data) and virtual world platforms (e.g., Second Life) differ conceptually from formal neuropsychological assessment, but they were included in this review due to their relevance to the lived experience and functional management of brain fog.

Social media analyses provide large-scale qualitative insight into how patients define and experience cognitive symptoms, revealing heterogeneity in symptom interpretation and overlap with dissociation, fatigue, and effort intolerance. These findings help clarify the phenomenological breadth of “brain fog,” supporting its multidimensional conceptualization.

Virtual environment studies demonstrated the feasibility of cognitively accessible social support platforms tailored to ME/CFS-specific limitations, indirectly addressing cognitive load and environmental overstimulation.

While these approaches do not constitute objective cognitive testing, they contribute to an understanding of cognitive burden, to digital accessibility, and to patient-centered design. Their inclusion is therefore justified within a broader management-oriented perspective rather than as a form of strict neuropsychological assessment.

### 3.7. Methodological Quality and Gaps

Across the included studies, the following are noted:

- Cross-sectional designs predominated.
- Sample sizes varied widely, from single-case studies to large EHR datasets.
- Few studies have conducted external validations of digital cognitive tools.
- Direct comparison between subjective and objective cognitive measures was rare.
- Longitudinal monitoring of brain fog variability remains underexplored.

Notably, although processing speed and sustained attention deficits showed the most consistent discrimination across these behavioral studies, integration of neurobiological, behavioral, and subjective data within a single digital framework was uncommon.

### 3.8. Overall Synthesis

Digital approaches to cognitive dysfunction in ME/CFS are evolving along two parallel trajectories: (1) objective mechanistic characterization using neuroimaging and computational phenotyping and (2) scalable behavioral and symptom-based monitoring via mobile and web platforms. While several tools demonstrate feasibility and discriminatory capacity, the field remains fragmented, with limited cross-validation, heterogeneous outcome definitions, and insufficient longitudinal evidence.

Future research should prioritize multimodal integration, standardized cognitive domain classification, and validation of digital tools specifically against the multidimensional construct of brain fog.

## 4. Discussion

This review examined the current evidence on digital approaches relevant to the assessment and management of cognitive dysfunction (“brain fog”) in ME/CFS. Across the 16 studies identified through a structured search, digital technologies were applied in heterogeneous ways, ranging from computerized neuropsychological testing and neuroimaging analytics to patient-generated data streams and digitally delivered interventions. When synthesized across these studies, three functional domains of digital application become apparent: (1) digital tools for cognitive assessment, (2) digital platforms for longitudinal monitoring and data integration, and (3) digitally mediated interventions or rehabilitation approaches. However, the evidence base remains uneven across these domains, with the strongest evidence concentrated in digital cognitive assessment and considerably fewer studies addressing monitoring or intervention.

### 4.1. Positioning of Digital Approaches Within the ME/CFS Literature

Cognitive dysfunction is widely recognized as a central and disabling feature of ME/CFS, and its clinical characterization has been addressed in several previous reviews of neuropsychological findings and pathophysiological mechanisms. However, these reviews have largely focused on traditional neuropsychological testing or neurobiological correlates, while the role of digital technologies in assessing and managing cognitive symptoms has received comparatively limited attention. The present review, therefore, contributes through a specific examination of how digital tools—ranging from computerized cognitive testing to real-time symptom tracking and digital therapeutics—are being applied in this domain.

The relatively small number of studies identified (n = 16) is notable when contrasted with the rapid expansion of digital health research as a whole. For example, exploratory Scopus database searches conducted without ME/CFS-specific terms indicated that publications combining digital technologies and cognitive assessment exceeded 23,000 records by the end of 2025. In fields such as neurodegenerative disorders, traumatic brain injury, and post-stroke rehabilitation, digital cognitive assessment platforms, mobile monitoring tools, and machine learning-based biomarkers are now widely studied. The comparatively limited adoption of such approaches in ME/CFS, therefore, highlights an important research gap rather than a lack of technological feasibility. Given that cognitive impairment and fluctuating mental fatigue are among the most frequently reported and functionally limiting symptoms of ME/CFS, the underrepresentation of digital cognition research in this population suggests substantial untapped potential for methodological and clinical innovation.

### 4.2. Evidence Across Categories of Digital Tools

When the identified studies were examined collectively, digital cognitive assessment tools emerged as the most mature and consistently applied category. Computerized neuropsychological tests, smartphone-based reaction time applications, and digital cognitive testing platforms were repeatedly able to detect differences between ME/CFS patients and healthy controls or to characterize specific domains of impairment, including attention, processing speed, and working memory. Importantly, several of these tools offer practical advantages compared with conventional paper-based testing, including standardized administration, automated scoring, and the possibility of remote assessment. These features are particularly relevant in ME/CFS, where patients often experience mobility limitations and post-exertional symptom exacerbation that complicate in-person clinical testing.

Neuroimaging studies incorporating advanced digital analytics represent a second line of evidence supporting the biological basis of cognitive dysfunction in ME/CFS. Techniques such as functional MRI combined with ML algorithms or quantitative perfusion imaging have revealed alterations in functional connectivity, regional cerebral blood flow, and multiregional brain activation patterns associated with fatigue severity and cognitive complaints. While these approaches remain largely confined to research settings due to their cost and technical complexity, they contribute to biomarker discovery and provide objective correlates of patient-reported cognitive difficulties.

A third emerging category involves patient-generated digital data, including that from symptom tracking applications, wearable sensors, and analyses of online patient narratives. These approaches offer complementary insights into the lived experience and temporal dynamics of brain fog, capturing fluctuations in cognitive effort, attentional capacity, and perceived mental fatigue in everyday environments. Social media analyses and digital diaries, although methodologically heterogeneous, highlight the multifaceted nature of brain fog and its contextual triggers. While such data sources are not equivalent to formal cognitive testing, they provide ecologically valid perspectives that may inform hypothesis generation and patient-centered outcome measures.

Finally, digital interventions targeting cognitive symptoms remain relatively underdeveloped. Only a small number of studies investigated digitally delivered therapeutic approaches, including online psychological interventions, virtual stress-management programs, or telerehabilitation protocols. These interventions generally focused on broader symptom management rather than directly targeting cognitive performance. Nevertheless, their results suggest that digital delivery formats may offer feasible and accessible modes of supporting patients, particularly when designed to accommodate the pacing strategies and energy limitations characteristic of ME/CFS.

### 4.3. Strength of Evidence and Methodological Limitations

Despite these promising developments, the methodological strength of the available evidence varies considerably. A number of studies involved small sample sizes or exploratory designs, limiting their statistical power and generalizability. Several digital approaches were evaluated in single-case studies or pilot cohorts, including multi-omics integration in an individual patient and exploratory therapeutic interventions. Such studies provide valuable proof-of-concept insights but should be interpreted cautiously and cannot establish clinical efficacy.

Similarly, some investigations remained protocol descriptions rather than completed trials, while others relied on cross-sectional observational data. Neuroimaging studies, although technically sophisticated, often involve modest sample sizes and may reflect highly selected patient populations. In addition, cognitive outcomes were assessed using heterogeneous instruments and metrics across the studies, complicating direct comparison or synthesis.

Another important limitation concerns validation and disease specificity. Many digital cognitive tools applied in ME/CFS research were originally developed for other neurological or psychological conditions and have not been specifically validated in ME/CFS populations. While this reflects a pragmatic reuse of available technologies, it also raises questions regarding measurement sensitivity, ecological validity, and interpretability in the context of fluctuating fatigue-related cognitive symptoms.

Taken together, these factors suggest that the current evidence base should be viewed as emerging and exploratory rather than a mature clinical evidence base. Future research would benefit from larger, longitudinal studies with standardized cognitive endpoints, rigorous validation of digital biomarkers, and the integration of digital measures with clinical and biological data.

### 4.4. Conceptualizing the Role of Digital Technologies in ME/CFS

One of the challenges in synthesizing the literature arises from the broad and heterogeneous use of the term “digital approaches.” In practice, the studies reviewed span a spectrum of technologies serving distinct purposes. Conceptually, these technologies may be positioned along a continuum from assessment, through monitoring, to intervention.

To facilitate interpretation of the heterogeneous literature, the findings of the included studies were conceptually synthesized into key domains reflecting the main roles of digital technologies in ME/CFS research and care (Table 2).

These domains illustrate how digital tools may support remote symptom monitoring, cognitive assessment, telehealth delivery, and patient-centered self-management. Digital cognitive tests and neuroimaging analytics primarily contribute to objective assessment and biomarker discovery. Patient-generated data streams and wearable sensors support the longitudinal monitoring and ecological measurement of symptom fluctuations. Digital therapeutic platforms and telerehabilitation programs represent early attempts to translate these insights into intervention and clinical management strategies.

Within this framework, it also becomes evident that many digital tools currently applied in ME/CFS research are borrowed from other fields, particularly cognitive neuroscience, mental health research, and neurorehabilitation. While such cross-disciplinary borrowing accelerates methodological development, it also underscores the need for ME/CFS-specific validation and adaptation, especially given the unique characteristics of post-exertional symptom exacerbation, cognitive fatigability, and fluctuating functional capacity.

### 4.5. Implications for Research and Clinical Practice

Digital technologies may offer several advantages in addressing longstanding challenges in ME/CFS research. Traditional clinic-based assessments often fail to capture the fluctuating and context-dependent nature of brain fog. In contrast, digital tools enable remote, repeated, and low-burden data collection, potentially allowing researchers to observe cognitive changes over time and in relation to physiological or environmental factors.

Furthermore, digital approaches may facilitate multimodal data integration, combining cognitive testing, physiological monitoring, patient-reported outcomes, and biological biomarkers. Such integrated datasets could support more refined phenotyping and contribute to the identification of clinically meaningful subgroups within the heterogeneous ME/CFS population.

However, implementation must also consider the specific needs and vulnerabilities of ME/CFS patients. Excessive digital monitoring, prolonged cognitive tasks, or poorly designed interfaces may exacerbate symptoms rather than alleviate them. Consequently, future digital tools should prioritize accessibility, a low cognitive load, pacing compatibility, and patient-centered design.

### 4.6. Limitations of the Present Review

Several limitations of this review should be acknowledged. First, the search strategy focused specifically on studies with explicit descriptions of digital tools in relation to cognitive outcomes, which may have excluded investigations where digital methods were used but not highlighted in the title, abstract, or keywords. This issue is illustrated by one of the authors’ previous studies, which reported the results of a large-scale online comparative survey of individuals with ME/CFS in Italy, Latvia, and the United Kingdom [40]; however, the digital modality of the data collection was not explicitly highlighted in the abstract or keywords. Consequently, such studies were not retrieved in the present search, underscoring a broader challenge related to the inconsistent reporting and indexing of digital research components in the literature.

Second, the included studies were highly heterogeneous in terms of their design, digital modalities, and outcome measures, precluding quantitative synthesis. Finally, although the review followed a structured search and screening process, it was intended as a descriptive mapping of an emerging field rather than a formal systematic review with meta-analysis.

### 4.7. Future Directions

Despite these limitations, the synthesis presented herein highlights several promising directions for future research. Priority areas include the standardization of digital cognitive assessment protocols, validation of disease-specific digital biomarkers, and development of patient-centered digital interventions that account for the energy limitations and fluctuating symptoms characteristic of ME/CFS.

Importantly, the findings of this review also suggest that the commonly used term “brain fog,” although originating from patient-reported experience rather than a single formally defined neuropsychological construct, captures a multidimensional aspect of cognitive dysfunction in ME/CFS. Rather than representing a discrete cognitive deficit, brain fog appears to reflect the interaction between subjective cognitive symptoms, measurable cognitive performance changes, and broader physiological and psychosocial factors. This observation highlights the importance of interpreting cognitive dysfunction in ME/CFS within a broader biopsychosocial framework rather than solely through a narrow pathophysiological lens.

**Figure 3:**
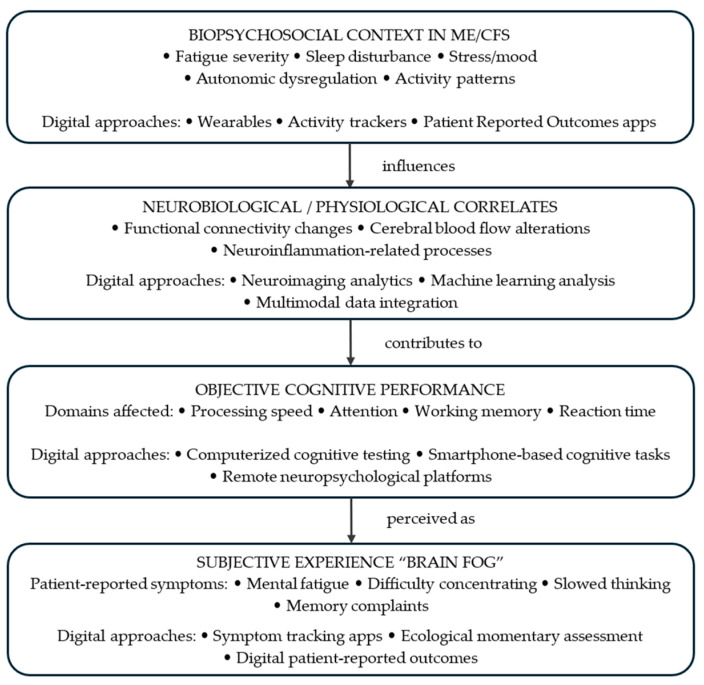
Biopsychosocial conceptualization of brain fog in ME/CFS and digital approaches used for its assessment and management.

The conceptual framework emerging from the present review is summarized in Figure 3, which illustrates the multidimensional pathway of brain fog in ME/CFS and the potential role of digital approaches in assessing and monitoring its different components.

As illustrated in Figure 3, brain fog in ME/CFS can be conceptualized as a dynamic phenomenon arising from interactions between neurobiological processes, objectively measurable cognitive performance alterations, and subjective cognitive symptoms within a broader biopsychosocial context. Digital technologies may provide useful tools for capturing these different dimensions through computerized cognitive testing, wearable monitoring, neuroimaging analytics, and patient-generated symptom data.

In the near term, digital tools may have their greatest impact in improving objective assessments and longitudinal monitoring of brain fog, thereby bridging the gap between patient-reported experiences and measurable clinical outcomes. Over time, the integration of digital cognitive metrics with biological, behavioral, and environmental data may support more precise phenotyping and personalized approaches to managing cognitive dysfunction in ME/CFS.

## 5. Conclusions

ME/CFS remains a high-burden yet under-researched condition, characterized by substantial diagnostic uncertainty, heterogeneous clinical manifestations, and a limited arsenal of evidence-based management strategies. The absence of validated biological biomarkers and disease-modifying treatments continues to impede timely diagnosis, effective management, and meaningful improvements in patients’ quality of life. Cognitive impairment, commonly described by patients as “brain fog,” represents one of the most disabling and pervasive manifestations of ME/CFS, yet it remains insufficiently addressed in both clinical practice and research.

This descriptive review focused specifically on digital approaches applied to the assessment and management of cognitive dysfunction in ME/CFS. The findings indicate that, despite the rapid expansion of digital health technologies across many areas of medicine, only a small proportion of the existing digital health literature has been directed toward ME/CFS, and an even smaller subset has addressed cognitive symptoms explicitly. Where digital tools have been applied, they have most often been used for objective cognitive assessment, neuroimaging-based analyses, or large-scale data integration, while digitally supported interventions and longitudinal cognitive monitoring remain scarce. These observations highlight a significant missed opportunity.

This study further suggests that brain fog, frequently reported by patients, can be viewed as a visible manifestation of the broader and multifaceted disease process underlying ME/CFS. Although not a single neuropsychological entity, the symptom may serve as an important clinical signal for earlier recognition of the condition and for selecting appropriate management strategies. Interpreting brain fog within a biopsychosocial framework may therefore enhance the potential of digital technologies to support more personalized monitoring and care.

Digital technologies offer unique advantages for ME/CFS, including the ability to capture symptom fluctuations in real-world settings, support remote and low-burden assessments, and integrate patient-reported outcomes with objective data. More systematic and targeted use of digital solutions could therefore contribute meaningfully to improved symptom characterization, personalized management strategies, and enhanced patient engagement. Future research should prioritize the validation of digital cognitive measures for ME/CFS, the development of disease-specific digital biomarkers, and the integration of digital tools into multidisciplinary care models. Harnessing the full potential of digital technology may represent an important step toward addressing longstanding gaps in ME/CFS management and improving outcomes for affected individuals.

## Figures and Tables

**Table 1:** Classification of digital approaches to cognitive dysfunction in ME/CFS according to cognitive domain, measurement level, and digital function.

Study	Digital Modality	Cognitive Domain(s)	Measurement Level	Digital Function	Primary Focus
McCue et al., 2002 [24]	CDR ^1^ computerized + telephone battery	Attention, Working Memory, Long-term Memory	Behavioral	Assessment	Objective deficits
Lange et al., 2005 [25]	BOLD ^2^ fMRI	Global cognitive dysfunction (neural correlates)	Neurobiological	Mechanistic	Validation of subjective difficulty
Best & Butler, 2014 [26]	Virtual environment (Second Life)	Cognitive fatigue (indirect)	Subjective/contextual	Social support	Functional adaptation
Maclachlan et al., 2017 [27]	REDCap ^3^ DSQ ^4^ + neuropsych tests	Verbal memory, Visuospatial memory, Processing speed, Working memory	Subjective + Behavioral	Assessment	Phenotype differentiation
Provenzano et al., 2020 [28]	ML ^5^ + fMRI	Neural pattern differentiation	Neurobiological	Mechanistic/ML	Biomarker classification
Li et al., 2021 [29]	PCASL ^6^ MRI (rCBF ^7^)	Brain perfusion associated with fatigue severity	Neurobiological	Mechanistic	Pathophysiology
Vernon et al., 2022 [30]	DANA ^8^ smartphone app	Reaction time (Processing speed)	Behavioral	Assessment (mobile)	Orthostatic challenge
Fernández-Quirós et al., 2023 [31]	CPT3™ ^9^ computerized test	Sustained attention, Response speed	Behavioral	Assessment	Diagnostic discrimination
McWhirter et al., 2023 [32]	Reddit scraping	Phenomenology of brain fog	Subjective	Phenomenological analysis	Lived experience
Jahanbani et al., 2024 [33]	App + sentiment + multi-omics	Cognitive fatigue (self-report)	Subjective + Digital integration	Monitoring/AI ^10^ integration	Personalized tracking
Lappalainen et al., 2024 [34]	Internet ACT ^11^	Global cognitive complaints (secondary)	Subjective	Intervention	Psychological distress
May et al., 2024 [35]	Virtual CBSM ^12^	Cognitive fatigue (indirect via PEM reduction)	Subjective	Intervention	Stress management
Duricka & Liu, 2025 [36]	BrainCheck + wearables	Executive function, Processing speed, Recognition memory	Behavioral + Physiological	Assessment pre/post intervention	Treatment response
Powers et al., 2025 [37]	EHR + ML phenotyping	Cognitive impairment (coded)	Digital phenotyping	ML classification	Disease comparison
Fricke-Comellas et al., 2025 [38]	Telerehabilitation	Cognitive fatigue (expected outcome)	Subjective (planned)	Intervention	Functional improvement
Azimi et al., 2025 [39]	BrainCheck + questionnaires	Processing speed, Executive function, Global complaints	Behavioral + Subjective	Assessment + biomarker correlation	Subtype identification

^1^ CDR—Cognitive Drug Research; ^2^ BOLD—Blood Oxygen Level Dependent; ^3^ REDCap—Research Electronic Data Capture; ^4^ DSQ—DePaul Symptom Questionnaire; ^5^ ML—Machine learning; ^6^ PCASL—Pseudo-continuous arterial spin labeling; ^7^ rCBF—Regional cerebral blood flow; ^8^ DANA—Defense Automated Neurobehavioral Assessment; ^9^ CPT3™—Continuous Performance Test; ^10^ AI—Artificial Intelligence; ^11^ ACT—Acceptance and Commitment Therapy; ^12^ CBSM—Cognitive Behavioral Stress Management.

**Table 2:** Conceptual synthesis of digital approaches for assessment and management of ME/CFS.

Conceptual Domain	Digital Approaches Identified in the Literature	Potential Clinical Value	Implementation Considerations for ME/CFS
Remote Symptom Monitoring	Mobile apps, wearable sensors, ecological momentary assessment, digital symptom diaries	Continuous tracking of fatigue, activity levels, sleep patterns, and symptom fluctuations; supports personalized monitoring and earlier identification of deterioration	Must minimize cognitive and physical burden; passive data collection preferable; interfaces should be simple and adaptable to fluctuating symptoms
Cognitive and Neurofunctional Assessment	Digital cognitive tests, app-based neuropsychological tasks, remote assessment platforms	Objective assessment of cognitive symptoms (e.g., attention, memory, processing speed) associated with “brain fog”	Tasks must be short, paced, and adaptable to avoid post-exertional symptom exacerbation; repeated measures may support monitoring of cognitive variability
Telehealth and Remote Care Delivery	Teleconsultations, telemonitoring platforms, remote multidisciplinary support	Improves access to specialist care for patients with mobility limitations; facilitates ongoing clinical monitoring and care coordination	Scheduling flexibility and pacing considerations are critical; clinicians should monitor for digital fatigue and symptom exacerbation
Digital Self-Management and Behavioral Interventions	Mobile health (mHealth) platforms, digital CBT-informed tools, pacing support applications	Supports symptom management strategies, pacing education, and patient self-management	Interventions must emphasize energy management rather than symptom pushing; careful design needed to avoid over-engagement or cognitive overload
Mind–Body and Cognitive Support Tools	Digital mindfulness, relaxation, or cognitive support exercises delivered via apps or online platforms	May support cognitive resilience, stress regulation, and symptom coping, potentially mitigating aspects of cognitive dysfunction	Sessions should be brief, optional, and adaptable to patient capacity; evidence base remains limited and requires further validation
Data Integration and Clinical Decision Support	Integration of wearable or patient-reported data into digital health platforms	Enables longitudinal symptom profiling and may assist clinicians in identifying triggers, pacing thresholds, or relapse patterns	Requires careful validation, privacy protection, and clinician training to interpret data meaningfully
Conceptual Domain	Digital Approaches Identified in the Literature	Potential Clinical Value	Implementation Considerations for ME/CFS
Remote Symptom Monitoring	Mobile apps, wearable sensors, ecological momentary assessment, digital symptom diaries	Continuous tracking of fatigue, activity levels, sleep patterns, and symptom fluctuations; supports personalized monitoring and earlier identification of deterioration	Must minimize cognitive and physical burden; passive data collection preferable; interfaces should be simple and adaptable to fluctuating symptoms
Cognitive and Neurofunctional Assessment	Digital cognitive tests, app-based neuropsychological tasks, remote assessment platforms	Objective assessment of cognitive symptoms (e.g., attention, memory, processing speed) associated with “brain fog”	Tasks must be short, paced, and adaptable to avoid post-exertional symptom exacerbation; repeated measures may support monitoring of cognitive variability
Telehealth and Remote Care Delivery	Teleconsultations, telemonitoring platforms, remote multidisciplinary support	Improves access to specialist care for patients with mobility limitations; facilitates ongoing clinical monitoring and care coordination	Scheduling flexibility and pacing considerations are critical; clinicians should monitor for digital fatigue and symptom exacerbation
Digital Self-Management and Behavioral Interventions	Mobile health (mHealth) platforms, digital CBT-informed tools, pacing support applications	Supports symptom management strategies, pacing education, and patient self-management	Interventions must emphasize energy management rather than symptom pushing; careful design needed to avoid over-engagement or cognitive overload
Mind–Body and Cognitive Support Tools	Digital mindfulness, relaxation, or cognitive support exercises delivered via apps or online platforms	May support cognitive resilience, stress regulation, and symptom coping, potentially mitigating aspects of cognitive dysfunction	Sessions should be brief, optional, and adaptable to patient capacity; evidence base remains limited and requires further validation
Data Integration and Clinical Decision Support	Integration of wearable or patient-reported data into digital health platforms	Enables longitudinal symptom profiling and may assist clinicians in identifying triggers, pacing thresholds, or relapse patterns	Requires careful validation, privacy protection, and clinician training to interpret data meaningfully
Conceptual Domain	Digital Approaches Identified in the Literature	Potential Clinical Value	Implementation Considerations for ME/CFS
Remote Symptom Monitoring	Mobile apps, wearable sensors, ecological momentary assessment, digital symptom diaries	Continuous tracking of fatigue, activity levels, sleep patterns, and symptom fluctuations; supports personalized monitoring and earlier identification of deterioration	Must minimize cognitive and physical burden; passive data collection preferable; interfaces should be simple and adaptable to fluctuating symptoms

## Data Availability

No new data were created or analyzed in this study.

## Abbreviations

The following abbreviations are used in this manuscript:

ACT	Acceptance and Commitment Therapy
AI	Artificial Intelligence
ATP	Adenosine triphosphate
AVLT	Auditory-verbal learning test
BOLD	Blood Oxygen Level Dependent
CBSM	Cognitive Behavioral Stress Management
CDR	Cognitive Drug Research
COGFAIL	Cognitive Failures Questionnaire
COMPASS	Composite Autonomic Symptom Score
CPs	Computable phenotypes
DSQ	DePaul Symptom Questionnaire
DSS	Digit Symbol Substitution
EHR	Electronic Health Record
FGF-21	Fibroblast growth factor 21
fMRI	Functional magnetic resonance imaging
GWI	Gulf War Illness
IDR	Immediate and Delayed Recognition
ME/CFS	Myalgic encephalomyelitis/chronic fatigue syndrome
ML	Machine learning
PASC	Post-acute sequelae of SARS-CoV-2 infection
PCASL	Pseudo-continuous arterial spin labeling
PCS	Post-COVID Syndrome
PEM	Post-exertional malaise
PPS	Persistent physical symptoms
PRISMA	Preferred Reporting Items for Systematic Reviews and Meta-Analyses
rCBF	Regional cerebral blood flow
REDCap	Research Electronic Data Capture
SGB	Stellate ganglion block
TAU	Treatment as Usual
Trails	Trail Making Test Parts A and B
VPT	Visual Patterns Test

## Appendix A

**Table A1:** Summary of the selected studies on digital approaches to managing cognitive impairments in ME/CFS, cognition assessment-related digital solutions, and cognition digital assessment-related results and conclusions.

	Authors, Year	Research Aim	Study Design	Number of Patients	Digital Solutions	Results and Conclusions
1	McCue et al., 2002 [24]	Assessment of the aspects of attention, working memory and long-term memory	A cohort study with a control group	30 CFS patients and 30 healthy controls	Computerized version of the CDR cognitive assessment test battery and a completely automated telephone version of the same battery	The findings of the study support the use of a fully automated telephone cognitive testing system for detecting deficits in CFS.
2	Lange et al., 2005 [25]	Evaluation of the auditory information processing difficulties	A cohort study with a control group	462 CFS patients and 226 controls	BOLD fMRI	The findings provide objective evidence for the subjective experience of cognitive difficulties in individuals with CFS.
3	Best & Butler, 2014 [26]	Examining the use of Second Life as a platform for social support by people with ME/CFS	Observational study	107 ME/CFS patients	Virtual environment, which experienced by numerous participants at once, who are represented within the space by avatars	The study allowed to present the ME/CFS patients with a new design that takes into account their specific requirements.
4	Maclachlan et al., 2017 [27]	Examining whether current CFS diagnostic criteria are identifying different disease phenotypes using the DSQ.	Case–control study	49 CFS subjects and ten matched, sedentary community controls, excluded for co-morbid depression	DSQ, available in the shared library of REDCap, hosted at DePaul University; Self-reported features: COMPASS and COGFAIL; Objective cognitive assessment: the Rey AVLT, the DSS test, Digit Span for working and short-term memory, Spatial Span for working and short-term dynamic visuospatial memory, and the VPT to assess short-term fixed visuospatial memory.	Self-reported autonomic and cognitive symptoms were significantly greater in CFS subjects compared to controls. Visuospatial memory, verbal memory and psychomotor speed were significantly different between DSQ subgroups
5	Provenzano et al., 2020 [28]	Uncovering objective biomarkers to lend credibility to criteria for diagnosis and differentiation of the GWI and CFS	Cohort study	80 GWI and 38 with CFS patients	ML algorithms used to assess a multi-regional pattern of differences in the fMRI scans	CFS and GWI are significantly differentiable using a pattern of fMRI activity based on an ensemble ML model.
6	Li et al., 2021 [29]	Investigation of the potential rCBF differences between ME/CFS patients and health controls; and association of the fatigue severity with the rCBF in the brain.	A cohort study with a control group	31 ME/CSF patients and 48 healthy controls	The PCASL technique on a whole-body clinical 3T MRI scanner. Besides routine clinical MRI, the protocol included a session of over 8 min-long rCBF measurement.	For the ME/CFS patients, the overall symptom severity score at rest was significantly associated with a reduced rCBF in the anterior cingulate cortex. The results of this study show that brain blood flow abnormalities in the limbic system may contribute to ME/CFS pathogenesis.
7	Vernon et al., 2022 [30]	Evaluation of orthostatic challenge and symptomatic, hemodynamic and cognitive responses in Long COVID and ME/CFS	A cohort study with a control group	42 Long COVID and 26 ME/CFS patients and 20 healthy controls	Cognition assessment by the Defense Automated Neurobehavioral Assessment (DANA) Brain Vital app for a patient’s smart phone, which rapidly evaluates an individual’s reaction time	The use of a smart phone app to assess cognition, can provide objective confirmation of the orthostatic intolerance and brain fog reported by patients with Long COVID and ME/CFS.
8	Fernández-Quirós et al., 2023 [31]	Delimiting the cognitive dysfunction associated with ME/CFS in adult patients by applying the Continuous Performance Test (CPT3^™^)	A cohort study with a control group	158 ME/CFS patients and 67 healthy controls	Computerized neuropsychological test CPT3^™^ to assess ME/CFS	The CPT3^™^ is a helpful tool to discriminate neurocognitive impairments from attention and response speed in ME/CFS patients, and it could be used as a marker of ME/CFS severity for diagnosing or monitoring this disease.
9	McWhirter et al., 2023 [32]	Examining first-person descriptions in order to better understand the phenomenology of brain fog	Observational study	1663 posts containing “brain fog” were identified, 717 meeting inclusion criteria	Posts containing “brain fog” were scraped from the social media platform Reddit, using Python V.3.9	“Brain fog” is used on Reddit to describe heterogeneous experiences, including of dissociation, fatigue, forgetfulness and excessive cognitive effort, and in association with a range of illnesses, drugs and behaviours.
10	Jahanbani et al., 2024 [33]	Integrating longitudinal multi-omics data with clinical, health, textual, pharmaceutical, and nutraceutical data	Case study	An extremely ill male ME/CFS patient	Patient blog post sentiment analysis; ME-CFSTrackerApp and LexiTime for real-time symptom tracking and enhance patient-physician/researcher communication, and evaluate response to medical intervention.	The study advocates for the integration of longitudinal multi-omics with multi-modal health data and AI techniques to better understand ME/CFS and its major comorbidities.
11	Lappalainen et al., 2024 [34]	Comparing a guided Internet-based ACT and TAU in reducing somatic complaints and psychological distress	3-month follow-up study of a randomized controlled trial	103 adults with PPS related to indoor environments, chronic fatigue or both conditions	Video-based case conceptualization + Internet-based ACT combined with TAU (iACT + TAU; n = 50) or TAU alone (n = 53)	Internet-based ACT, when combined with TAU, demonstrated efficacy for individuals with PPS associated with indoor environments and chronic fatigue.
12	May et al., 2024 [35]	Testing a virtual CBSM intervention	Randomized controlled trial	71 classified as highPEM, and 79 lowPEM ME/CFS patients	Comparing 10-week videoconference-delivered group CBSM (V-CBSM, n = 75) to a 10-week Health Information active control (V-HI, n = 75)	V-CBSM demonstrates benefits for ME/CFS patients presenting with severe PEM and may reduce the expression of PEM over time.
13	Duricka & Liu, 2025 [36]	Determining the effect of SGB on symptoms of ME/CFS	A prospective cohort pilot study	10 ME/CFS patients	Online BrainCheck cognitive testing platform. Neurocognitive assessment tests included Trails, Stroop Interference, DSS, and IDR. The SleepImage^®^ ring device collected electrocardiogram, actigram, plethysmogram, and oxygen saturation data, to measure sleep parameters.	Symptoms of ME/CFS were reduced after treatment with SGBs in this small prospective cohort pilot study. Objective measures of cognitive impairment were reduced following treatment, most notably in the areas of IDR.
14	Powers et al., 2025 [37]	Evaluation of similarities and differences between post-acute sequelae of PASC and ME/CFS	Electronic health record (EHR) cohort study	EHR data from 6.5 million adult patients: PASC (n = 28,318); ME/CFS (n = 152,205); Control group (n = 6,396,760)	EHR data integrated and harmonized in the N3C Data Enclave. ML-driven CPs.	Dyspnea, fatigue, and cognitive impairment are the most predictive conditions shared by both CPs. The cardiac and respiratory conditions are more typical for PASC. Records of pain, sleep disturbances, and neuropsychiatric conditions more commonly accompany ME/CFS.
15	Fricke-Comellas et al., 2025 [38]	Comparing the effects of a telerehabilitation program based on conscious movement with those of conventional low-intensity exercise	Protocol for a prospective, single-blind, three-arm, randomized clinical trial	147 participants (aged 18–70) with ME/CFS or PCS will be recruited	Exercise-based telerehabilitation program	Conscious movement, which integrates exercise with interoception and mindfulness-based strategies, may provide greater benefits than low-intensity exercise alone.
16	Azimi et al., 2025 [39]	Evaluation of the Fibroblast growth FGF-21, a stress-responsive metabolic hormone, as a promising avenue to distinguish subtypes within ME and fibromyalgia patient populations	A cohort study with a control group	250 patients (ME = 99; FM = 47; ME + FM = 104) and 54 healthy controls	Symptoms burden and cognitive function were assessed using validated questionnaires (SF-36, MFI-20, DSQ, DPEMQ) and the BrainCheck platform	The findings suggest that FGF-21 may serve as a valuable biomarker for identifying clinically meaningful subtypes within ME and FM, supporting the development of personalized treatments.
